# Between Sensitivity and Specificity – an analysis of the relationship between the clinical picture, diagnostic delays and co-morbidity among adolescents with autism spectrum disorders

**DOI:** 10.1192/j.eurpsy.2025.453

**Published:** 2025-08-26

**Authors:** K. M. Wilczyński, A. Stasik, L. Cichon, I. Jelonek, M. Janas-Kozik

**Affiliations:** 1 Department of Psychiatry and Psychotherapy of Developmental Age, Medical University of Silesia, Katowice; 2 Department of Psychiatry and Psychotherapy of Developmental Age, John Paul 2nd Child and Family Health Center, Sosnowiec, Poland

## Abstract

**Introduction:**

Early diagnosis of ASD a very important factor in improving the quality of life of people on the spectrum. Isolated reports that analyse the effect of age of diagnosis on the comorbidity indicate a statistically significant relationship between these parameters. However, the literature on this subject is very poor and does not take into account the risk of self-aggression and suicide in this context.

**Objectives:**

In this two-stage study we have aimed to analyse the risk of comorbidity, specific diagnoses, and the risk of autoagression and suicidality depending on the age of diagnosis of ASD as well as we have tried to answer the question of what factors affect the age of diagnosis in Poland.

**Methods:**

First stage of the study was carried out in the form of a retrospective analysis of the documentation and, in total, included the documentation of 328 people who comprised all patients with ASD diagnosis hospitalised in inpatient and diagnosed in outpatient clinic of our Department in 2021 and 2022. Subsequently in the second stage 77 children were randomly recruited among the patients with the diagnosis of ASD who came to local mental health clinic at the Child and Family Health Center in Sosnowiec for the more detailed analysis.

**Results:**

In the first stage the mean age of diagnosis of ASD in the overall group was 11.28 years. The late diagnosis of ASD was also statistically significantly associated with the risk of psychiatric comorbidity. Furthermore in the logistic regression analysis, each subsequent year of diagnosis delay was statistically significantly associated with the risk of suicidal ideation with OR=1.29 (95%CI: 1.17-1.42; p<0.000001). Statistically significant parameters shaping the time of ASD diagnosis were: mutual communication, the ability to establish peer relationships and the appearance of atypical social behaviours. In the second stage of the study the mean age of the first enrollment in the mental health clinic was 9.09 years for girls and 6.42 for the boys. The age of the first visit to mental health clinic was correlated only with the total score of the ADOS-2 study (rho = -0.32; p = 0.0092) and the ADOS-2 social affect subscale (rho = -0.29; p = 0.012). Meanwhile, the time it took for a specialist to diagnose ASD depended on the TAS-20 score (rho = -0.30; p = 0.0049).

**Conclusions:**

The results of the study indicate that the diagnosis of ASD is still a significant problem both in Poland and in the world. The long time needed to obtain the correct diagnosis is a common problem. Furthermore delaying the correct diagnosis has a significant negative impact on function, health and prognosis in patients with ASD in many contexts. Inter allia in the analysed group it was responsible for a significant increase in risk of suicidal ideations.

**Disclosure of Interest:**

None Declared

